# Pre-exposure immunohematologic features of heart failure associate with COVID-19 mortality

**DOI:** 10.1038/s44325-024-00025-7

**Published:** 2024-11-21

**Authors:** David A. Zidar, Brigid M. Wilson, Sadeer G. Al-Kindi, David Sweet, Steven Juchnowski, Lauren Huntington, Carey Shive, Jürgen Bosch, Christopher King, Jonathan Karn, Mina K. Chung, Carl B. Gillombardo, Mohammad Karnib, Varun Sundaram, Sahil A. Parikh, Mukesh Jain, Douglas D. Gunzler, Jacek Skarbinski, W. H. Wilson Tang, Donald D. Anthony, Timothy A. Chan, Jarrod E. Dalton

**Affiliations:** 1 Case Western Reserve University School of Medicine, 10900 Euclid Ave, Cleveland, OH 44106 USA; 2 Louis Stokes Cleveland Veterans Affairs Medical Center, 10701 East Blvd., Cleveland, OH 44106 USA; 3 Harrington Heart and Vascular Institute, University Hospitals Cleveland Medical Center, 11100 Euclid Ave, Cleveland, OH 44106 USA; 4 Heart Vascular and Thoracic Institute, Cleveland Clinic, 9500 Euclid Avenue, Cleveland, OH 44195 USA; 5 Center for Interventional Vascular Therapy, Division of Cardiology, Columbia University Irving Medical Center, 161 Fort Washington Avenue, New York, NY 10032 USA; 6 Department of Molecular Biology, Cell Biology and Biochemistry, The Warren Alpert Medical School of Brown University, 222 Richmond St, Providence, RI 02903 USA; 7 Center for Healthcare Research and Policy, Case Western Reserve University at MetroHealth, 2500 MetroHealth Drive, Cleveland, OH 44109 USA; 8 Division of Research, Kaiser Permanente Northern California, 275 West MacArthur Boulevard, Oakland, CA 94611 USA

**Keywords:** Immunology, Health care

## Abstract

Chronic heart failure, like diabetes, is a pro-inflammatory cardiometabolic condition, but its association with immunodeficiency is less well established. We conducted a retrospective cohort study of US Veterans infected during the first wave of COVID-19 (*n* = 92,533) to identify relationships between comorbidities, pre-infection immunohematologic (IH) features (based on complete blood cell count parameters), and 60-day mortality. A biomarker sub-analysis of anti-SARS CoV2 antibodies and cytokine levels was also performed (*n* = 44). Heart failure was independently associated with higher COVID-19 mortality and with the specific IH alterations (especially relative anemia, anisocytosis, and lymphopenia) which themselves predicted non-survival or protracted inflammation. Over half the risk conferred by heart failure was mediated by its anticipatory IH features whereas diabetes risk was unrelated to its associated IH profile. These findings indicate that heart failure is associated with a COVID-19 immunodeficiency distinct from that of diabetes which correlates with antecedent erythrocyte and lymphocyte dyshomeostasis.

## Introduction

The diversity of symptoms and outcomes after SARS-COV2 exposure highlights the existence of substantial heterogeneity in immunologic function within adult populations. Most immuno-profiling efforts are currently impractical to scale to the general population, and thus the identification of those who may lack resilience prior to exposure remains difficult. Instead, demographics and clinical co-morbidities alone have generally been used to guide preventive and therapeutic guidelines^1^.

Some, but not all reports have suggested heart failure and/or cardiovascular disease risk factors are linked to lower survival among hospitalized adults with SARS-COV2 infection^2–7^. This is plausible given the proposed relationships between cardiometabolic disease and immune dysfunction^8,9^. However, the extent to which relationships between heart failure and COVID-19 outcomes are clouded by differences in demographic characteristics, associated risk factors such as diabetes, or latent processes such as immune dysfunction has not been definitively addressed.

Parameters routinely reported via complete blood cell (CBC) counts can reflect disruptions in immune and/or hematologic homeostasis and, albeit crude, associate with the risk of adverse cardiovascular and non-cardiovascular events^10–12^. These measures are broadly accessible, low cost, and therefore scalable to populations.

Here, we sought to test the hypothesis that chronic heart failure is independently linked to COVID-19 mortality, in part due to associated immunohematologic (IH) dysfunction as identified by features in the complete blood cell count present prior to infection. In a large population, we analyze those with and without prevalent heart failure to identify its independent contribution to COVID-19 mortality and its association with immunohematologic alterations. We compare heart failure to diabetes given that the latter is a bono fide immunocompromised condition, each has been shown to be associated with inflammation, and both conditions are share certain pathogenic features (i.e. oxidative stress, mitochondrial dysfunction) and therapeutics (i.e. sodium glucose cotransporter 2 inhibitors, glucagon-like peptide 1 receptor agonists)^13,14^.

## Results

### Heart failure and diabetes each independently associate with increased COVID-19 mortality

92,533 patients were analyzed in the primary analysis (Table 1). The mean age was 62.4 (standard deviation, SD = 15.7). 10% (*N* = 9161) were female consistent with the VHA population, 32.5% were non-white, and 10.3% were Hispanic. Overall, 6605 patients died within 60 days of positive SARS-COV2 testing (7.1%). Those that died were typically older, less likely to be female, and had higher rates of various comorbidities including heart failure (24.7% versus 9.3% among survivors) and diabetes (55.2% versus 35.7% among survivors).

**Table 1:** Demographic and clinical characteristics

Characteristic		Total	Survivors	Non-survivors
N=		92533	85928	6605
Age (Median [IQR])		65.0 (51.8, 73.5)	63.7 (50.8, 72.9)	75.9 (71.2, 84.8)
Sex (% Female)		9.9%	10.5%	2.0%
Race	White	67.5%	67.4%	69.9%
	Black	23.3%	23.5%	21.1%
	American Indian	1.2%	1.2%	1.6%
	Unknown	6.9%	6.9%	6.7%
Ethnicity	Not Hispanic	86.4%	86.2%	88.9%
	Hispanic	10.3%	10.5%	8.0%
	Unknown	3.3%	3.3%	3.1%
Diabetes Mellitus		37.1%	35.7%	55.2%
Tobacco Smoking		62.6%	61.8%	73.2%
Prior Myocardial Infection		3.8%	3.4%	7.9%
Prior Heart Failure		10.4%	9.3%	24.7%
Prior Pulmonary Disease		22.7%	21.9%	33.6%
Prior Rheumatologic Disease		2.3%	2.2%	3.6%
Prior Cancer		12.9%	12.1%	23.6%
Statin Prescription		47.4%	46.6%	57.8%
Body Mass Index		30.6 (26.8, 43.8)	30.6 (27.0, 34.9)	28.7 (24.7, 33.5)
Systolic Blood Pressure		131 (120, 143)	131 (120, 142)	130 (117, 145)
Diastolic Blood Pressure		78 (70, 78)	78 (71, 85)	73 (65, 80)
Total Cholesterol		165 (138, 196)	167 (139, 197)	147 (123, 177)
Low Density Lipoprotein		94 (70, 121)	95 (71, 122)	79 (59, 103.8)
Serum creatinine		1.03 (0.90, 1.22)	1.02 (0.90, 1.20)	1.2 (0.99, 1.60)
Blood Urea Nitrogen		16 (12, 20)	16 (12, 20)	20 (15.0, 28.0)
Eosinophils		0.19 (0.10, 0.26)	0.19 (0.10, 0.25)	0.2 (0.10, 0.30)
Basophils		0.04 (0.00, 0.08)	0.04 (0.00, 0.80)	0.04 (0.00, 0.09)
Neutrophil Count		4.0 (3.2, 5.0)	4.0 (3.2, 4.9)	4.4 (3.5, 5.6)
Monocytes		0.60 (0.48, 0.70)	0.59 (0.47, 0.70)	0.6 (0.5, 0.78)
Lymphocytes		1.8 (1.4, 2.2)	1.8 (1.4, 2.3)	1.6 (1.2, 2.0)
Hemoglobin		14.2 (13.0, 15.2)	14.3 (13.1, 15.2)	13 (11.5, 14.4)
Mean Corpuscular Volume		90.2 (87.1, 93.2)	90.1 (87.0, 93.0)	91.4 (88.2, 95.5)
Red Cell Distribution Width		13.4 (12.8, 14.2)	13.4 (12.8, 14.2)	14.1 (13.3, 15.1)
Platelets		226 (189, 267)	227 (191, 268)	208 (168, 254)
Mean Platelet Volume		9.97 (9.3, 10.5)	9.98 (9.3, 10.5)	9.93 (9.4, 10.6)

**Fig. 1:**
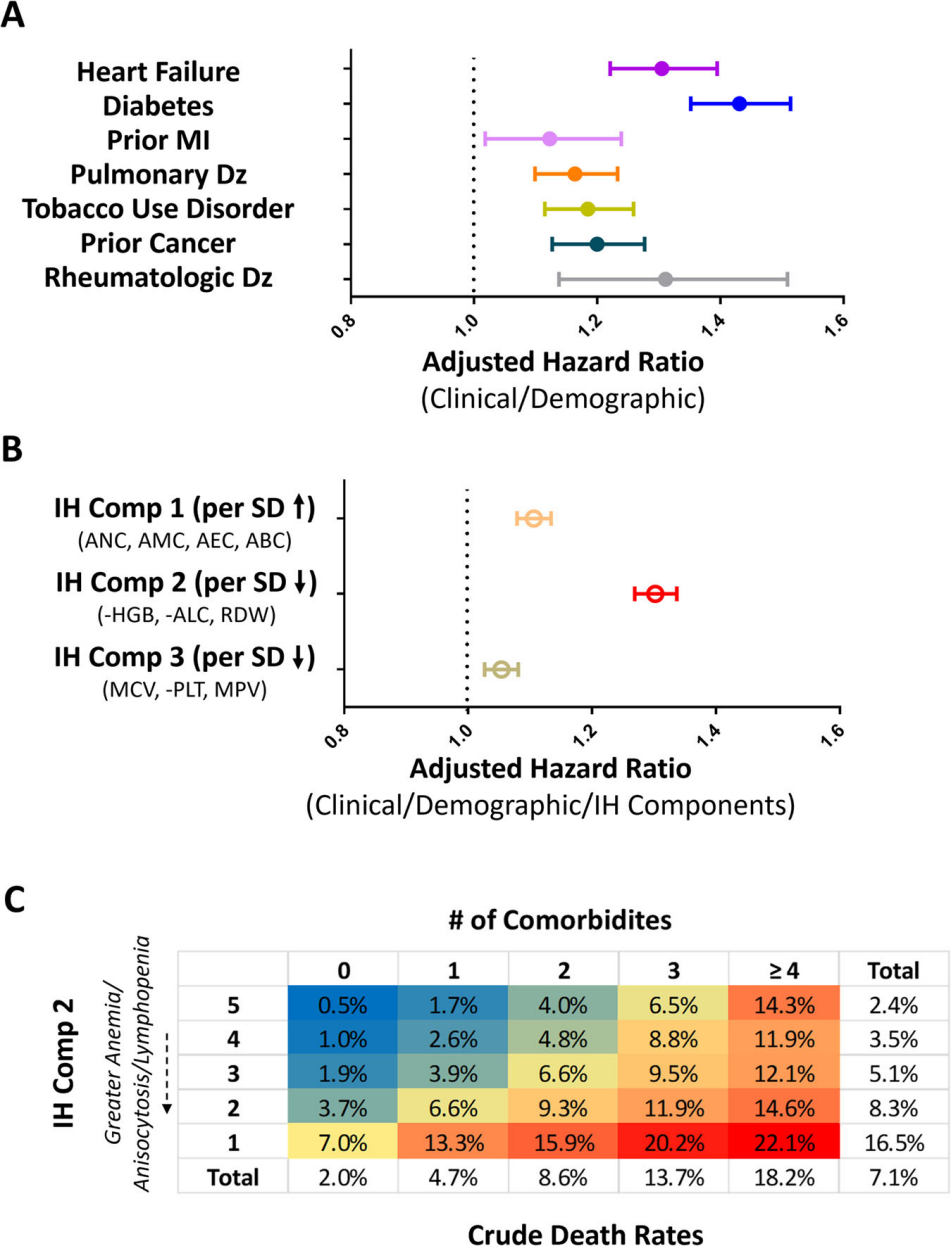
Antecedent comorbidities and immunohematologic variance are associated with COVID-19 mortality. **A** Adjusted hazard ratios (HRs) and 95% confidence intervals from cox-proportionate hazard models depict the associations between antecedent comorbidities and COVID-19 mortality after adjustment for demographic and clinical features. **B** Adjusted HRs depict the associations between antecedent immunohematologic (IH) components (per standard deviation). **C** Crude COVID-19 mortality rates (as percentages and heat map: blue- low mortality, red- high mortality) in relation to the quintile of Immunohematologic component 2 (rows) and the number of comorbidities (columns). (Comp: component; SD: standard deviation; MI: myocardial infarction; Hemoglobin: Hgb; Mean corpuscular volume: MCV; Red cell distribution width: RDW; Absolute neutrophil counts: ANC; Absolute monocyte counts: AMC; Absolute lymphocyte counts: ALC; Absolute basophil counts: ABC; Absolute eosinophil counts: AEC; platelet counts: Plt; mean platelet volume: MPV).

In univariable models; models adjusted for age, sex, race and ethnicity only; and models adjusted for all 20 clinical features; antecedent comorbidities were generally associated with increased risk of COVID-19 mortality (Fig. 1A and Supplementary Table 1). For instance, heart failure was associated with an unadjusted hazard ratio for COVID-19 mortality of 3.16 (95% confidence interval [CI]: 2.98–3.35), 1.66 (CI: 1.56–1.76) after adjustment for demographics alone, and 1.30 (CI: 1.22–1.39) in fully adjusted models. Diabetes was associated with an unadjusted odds ratio of 2.17 (CI: 2.06–2.28) for COVID-19 mortality, 1.55 (CI: 1.47–1.64) after adjustment for demographics alone, and 1.43 (CI: 1.35–1.51) in fully adjusted models. Prior MI (fully adjusted odds ratio [aOR]: 1.12 [CI: 1.02–1.24]), pulmonary disease (aOR: 1.16 [CI: 1.10–1.23]), tobacco use disorder (aOR: 1.19 [CI: 1.12–1.28]), cancer (aOR: 1.20 [CI: 1.13–1.28]), and rheumatologic disease (aOR: 1.31 [CI: 1.14–1.51]) also retained independent associations with COVID-19 mortality.

### Certain immunohematologic features anticipate COVID-19 mortality

By principal components analysis, over half of the heterogeneity among the 10 individual parameters was explained by 3 dominant (eigenvalues > 1) components (Supplementary Table 2). Component 1 explained over 20% of overall variation in CBC data and reflected the co-expansion of several leukocyte subsets, especially neutrophils, monocytes, and lymphocytes. Component 2 accounted for 16% of the overall variation and reflected associated changes in hemoglobin, RDW, and lymphocyte levels such that a lower IH component 2 signifies shared anemia, anisocytosis, and lymphopenia (AAL). Component 3 was predominantly related to variance of the MCV, platelet counts, and the MPV.

Several individual IH parameters retained independent associations with COVID-19 death in models adjusted for demographic and clinical features **(**Supplementary Table 3), but IH component 2 (i.e. AAL) was the strongest IH predictor (Fig. 1B). Specifically, in models adjusting for demographics, clinical features, and the other IH components, the shared anemia, anisocytosis, and lymphopenia reflected as a decline in IH component 2 was independently associated with a 30% increase in COVID-19 mortality (adjusted hazard ratio [aHR] per SD: 1.30; 95% CI: 1.34–1.27). This relationship held in females (aHR per SD: 1.51; 95% CI: 1.81–1.25) and in racial (Black: aHR: 1.23; 95% CI: 1.30–1.16) and ethnic (Hispanic: aHR: 1.20; 95% CI: 1.33–1.09) minorities. In addition to AAL, variance in IH component 1 (aHR per SD: 1.11; 95% CI: 1.08–1.13) and IH component 3 (aHR per SD: 0.95; 95% CI: 0.93–0.97) prior to infection was also independently associated with COVID-19 mortality, albeit more modestly than IH component 2.

According to crude mortality rates, each quintile of AAL was roughly equivalent to the risk conferred by one clinical co-morbidity (Fig. 1C). Similar results (Supplementary Fig. 1A) were observed in analyses using hemoglobin, RDW, or lymphocyte levels individually. In sex-stratified analyses, men and women between the ages of 40–60 years old who were in the lowest AAL quintile (highest risk) had a similar mortality rate as those 60–80 years of age who were in the highest AAL (most favorable) quintile (Supplementary Fig. 1B).

**Fig. 2:**
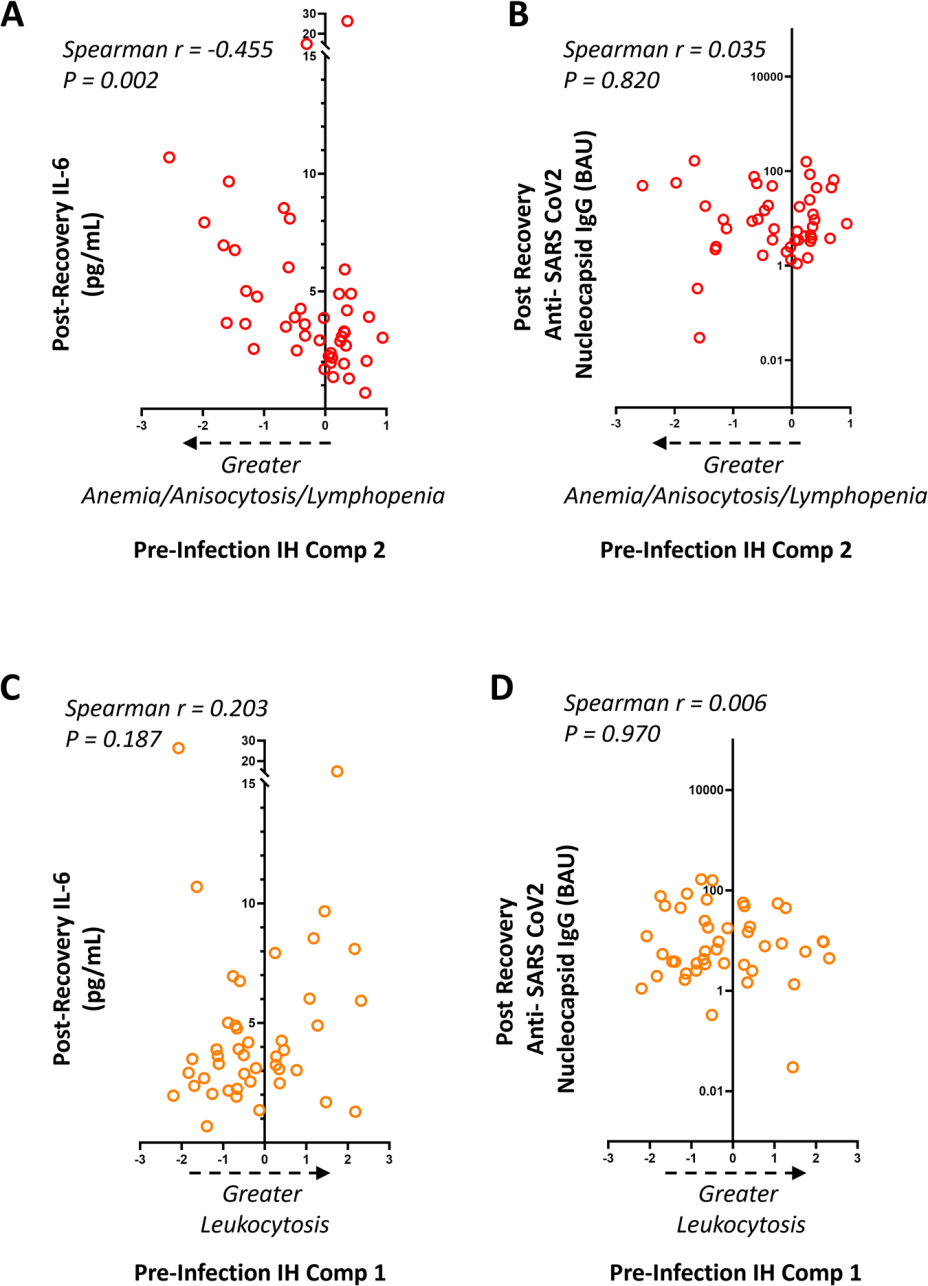
Pre-COVID-19 immunohematologic (IH) features correlate with inflammation after COVID-19 among survivors. Post recovery plasma IL-6 levels (**A**, **C**) and anti-nucleocapsid SARS- CoV2 antibody titers (**B**, **D**) from COVID-19 survivors are shown in relation to pre-infection IH components 1 and 2. (NCP: nucleocapsid; RBD: receptor binding domain; IFN: interferon; IL: interleukin; TNF: tumor necrosis factor).

In a subset with post-recovery blood analysis (*N* = 44; mean age: 68 year, 11% female, 41% Black, 5% Hispanic, mean time from infection = 425 days, range 635 to 330 days; see Supplementary Table 4 for full characteristics), pre-infection AAL correlated with higher IL-6 levels after recovery (rho = −0.455, *p* = 0.002), but was not related to the levels of anti-SARS-CoV2 nucleocapsid antibody levels (rho = 0.035, *p* > 0.05: Fig. 2A, B). The relationship between IL-6 and AAL remained significant after adjustment for demographics and time from COVID-19 (standardized beta −0.365, *p* = 0.04). In contrast, pre-infection IH component 1 did not correlate with post-recovery IL-6 or anti-nucleocapsid antibodies (Fig. 2C, D). In addition to IL-6, multiple other cytokines including TNFα, IL-17A, IL-21, IL-29, IL-4, IL-33, and IL-22 also had significant correlation with pre-infection AAL, but none were related to IH components 1 and 3 (Supplementary Fig. 2).

### Heart failure is associated with immunohematologic variation relevant to COVID-19 survival

Prevalent heart failure was associated with significant alterations in IH parameters and components, prior to SARS-CoV2 infection (Supplementary Table 5). Among individual parameters and components, AAL (i.e. decreased IH Component 2) was the parameter most closely associated with prevalent heart failure (aOR= 0.61 [0.59–0.62] per SD IH component 2). This relationship was similarly strong regardless of whether patients exhibited heart failure with preserved or reduced ejection fraction (Supplementary Fig. 3). In comparison to IH component 2, pan-leukocytosis as captured in IH component 1 (aOR = 1.13 [1.11–1.16] per SD IH component 1) and platelet parameters and red cell size as depicted in IH component 3 (aOR = 0.82 [0.80–0.84] per SD IH component 3) were far weaker as correlates of heart failure.

**Fig. 3:**
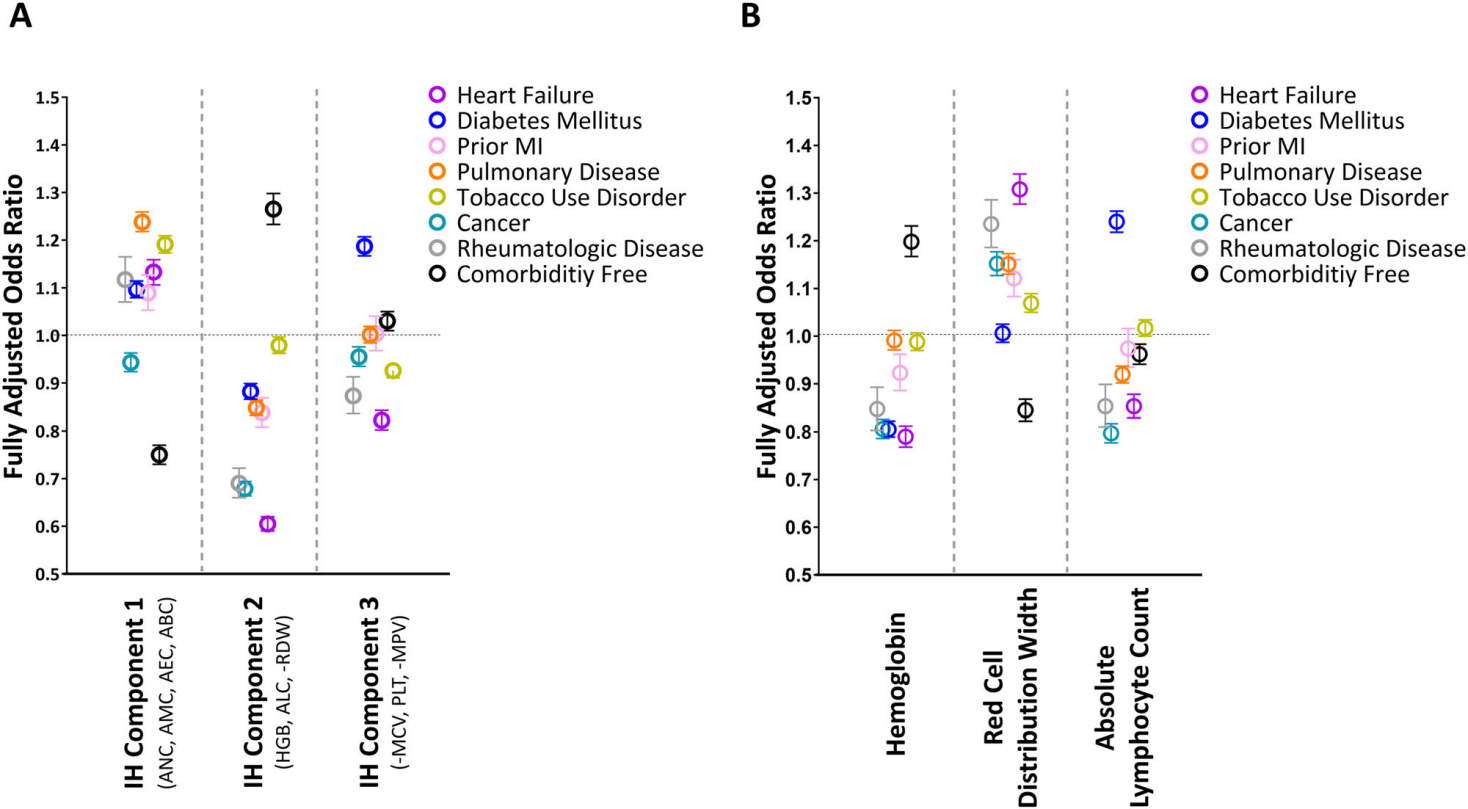
Comorbidities are associated with differing patterns of immunohematologic (IH) variance in advance of COVID-19. **A** In models fully adjusted for demographics, clinical, and other IH components, the odds ratio (with 95% confidence intervals) for the co-morbidity shown is illustrated per standardized unit increase for a given IH component. **B** The odds of having each co-morbidity is illustrated per standardized unit increase in the individual CBC parameter. (Absolute neutrophil counts: ANC; Absolute monocyte counts: AMC; Absolute lymphocyte counts: ALC; Absolute basophil counts: ABC; Absolute eosinophil counts: AEC; Hemoglobin: HGB; Mean corpuscular volume: MCV; Red cell distribution width: RDW; platelet counts: PLT; mean platelet volume: MPV; MI: myocardial infarction).

Compared with other co-morbidities, heart failure was the condition most strongly associated with AAL (Fig. 3A). In contrast, diabetes was associated with similar increases in IH component 1, but only a moderate decline in IH component 2, and an elevated IH component 3. In fact, although diabetes was associated with a similar degree of anemia, it was characterized by a relative lymphocytosis instead of lymphopenia and less anisocytosis compared to heart failure (Fig. 3B).

Given this relationship between AAL and heart failure, and that each were antecedent predictors of COVID-19 mortality, we sought to quantify the extent to which the risk assigned to heart failure could be explained by its latent relationship with AAL. Mediation analyses, fully adjusting for demographic and clinical features, suggested a substantial portion of the association between heart failure and COVID-19 mortality was explained by its associated pre-infection immunohematologic milieu (Supplementary Table 6). Specifically, pre-infection IH features were responsible for 53.2% of the mortality risk associated with heart failure (effect of all 3 IH components: beta = 0.149 [0.131–0.165], *p* < 0.001; non-IH related effects: beta = 0.131 [0.058–0.205], *p* < 0.001), and this was almost exclusively due to AAL (IH Component 2: beta = 0.132 [0.116–0.147], *p* < 0.001). In contrast, IH features explained less than 10% of the risk associated with diabetes (effect of all 3 IH components: beta = 0.030 [0.024–0.037], *p* < 0.001; non-IH related effects: beta = 0.343 [0.284–0.402], *p* < 0.001).

Taken together, these analyses show that AAL can serve as a correlate of infectious risk, is especially prevalent in those with chronic heart failure, and helps to explain its associated COVID-19 mortality risk. Moreover, these data also suggest that diabetes, which also confers substantial excess COVID-19 mortality risk, does so via mechanisms that are poorly identified by CBC parameters.

## Discussion

In this large cohort of patients who experienced SARS-CoV2 infection during the first wave of COVID-19, antecedent chronic heart failure was associated with increased COVID-19 mortality, and this relationship held after adjustment for a wide variety of demographic and clinical factors. Moreover, we find certain immunohematologic features, especially anemia/anisocytosis/lymphopenia- AAL, were independently related to COVID-19 mortality as well as prevalent heart failure. These immunohematologic features explained approximately half the COVID-19 mortality risk associated with heart failure and correlated with cytokine elevation among recovered survivors. In contrast, diabetes was associated with similar or greater COVID-19 risk but with minimal relation to its antecedent immunohematologic features.

This is among the largest examination of prevalent cardiovascular disease and COVID-19 mortality published to date. Heightened infection risk related to diabetes is well accepted but whether heart failure is also an immunocompromising condition has been less well established. Some^5,6^ but not all^7^ previous studies have identified heart failure to be a univariable predictor of death in hospitalized patients with COVID-19^15^. Here, we sought to first validate this association in a sample size ~20 times larger than most previous studies and after adjustment for a broad range of potential confounders. We conclude that even after rigorously accounting for clinical confounders, prevalent heart failure independently increases the mortality risk associated with SARS-CoV2 infection. These results support the efforts to minimize infectious exposures, maximize vaccination rates, and understand the mechanisms of immunologic risk in those with heart failure. Additional studies are also needed to understand the extent to which this infectious risk (and the IH correlates thereof) is mitigated by the medical therapies which improve heart function.

This is the first comprehensive analysis of *pre-exposure* immunohematologic characteristics, how they relate to prevalent co-morbidities, and associate prospectively with COVID-19 survival. Our data indicate the prognostic importance of baseline immunohematologic variance to be substantial, even when assessed as crude biomarkers available in CBC results. This latent IH risk was substantial in magnitude, even after adjusting for known co-morbidities. In particular, AAL (i.e. IH component 2 reductions) was especially powerful as a predictor. Each quintile of IH component 2 roughly equated to the risk equivalent of one comorbidity. Membership in the lowest versus highest quintile was equivalent to 10- 20 years of aging on the basis of COVID-19 mortality risk. Variance in other CBC parameters such as IH component 1, indicative of general elevation of leukocytes, was a more moderate indicator of heighten risk which was additive to that conferred by AAL.

The use of PCA provides a data reduction step, consolidating 10 variables to 3, but was also employed to capture underlying biologic processes that may affect several CBC parameters. Thus, IH component 1 seems to reflect the production and marginalization of leukocytes in general whereas IH component 2 (AAL) captures the tendency for inflammation to negatively impact red cell (i.e. inflammation associated anemia) and T cell homeostasis. Our data argues that the latter is stronger than the former as an anticipatory surrogate of COVID-19 risk.

We find that pre-infection AAL strongly correlated with elevated IL-6, TNFα, and other cytokines months or years after infection. This is consistent with previous reports by our group and others showing anisocytosis to be associated with a pro-inflammatory milieu^16^. The RDW has also been shown to be highly predictive of mortality in those with prevalent heart failure in association with IL-6 levels^12,17^. Of note, TNFα is known to be expressed by cardiomyocytes in the setting of heart failure^18^, is associated with endothelial dysfunction and myocardial fibrosis^19^, impairs erythropoiesis^20,21^, and links to T cell apoptosis^22–25^. In the setting of rheumatoid arthritis, we have shown lymphopenia can be reversed upon initiation of TNFα inhibitors^26^. Thus, our data extend this line of investigation to include the general population with or without heart failure, suggesting red cell homeostasis may reflect critical inflammatory processes which would otherwise be undetected by total leukocyte levels.

**Fig. 4:**
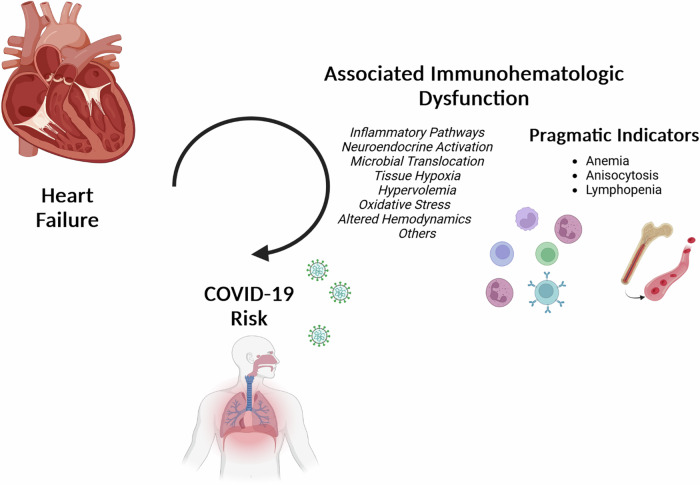
Immunohematologic alterations prevalent in heart failure identify latent COVID-19 mortality risk. Pragmatic indicators of excess COVID-19 risk (anemia, anisocytosis, and lymphopenia) are especially prevalent in heart failure and explain the majority of its associated risk. Interrupting the mechanisms through which heart failure impacts red cell and lymphocyte homeostasis could improve immunologic resilience in the general population as well as those living with heart failure.

That IH component 2 included both anisocytosis and lymphopenia is consistent with our previous observation that the mortality associated with subclinical lymphopenia is not only independent of, but is synergistic with an elevated RDW^10^. Thus, variance in IH component 2 as observed in this study may reflect mechanisms which directly and indirectly affect erythropoiesis as well as T cell homeostasis (apoptosis, replication, and/or re-circulation). In addition to TNF elevation, heart failure has been associated with reductions in IL-7 (relevant to T cell replication), altered expression of components of the CCR7 system, and the regulation of chemokine receptors in general via increases in G-protein coupled receptors kinases (relevant to trafficking). Thus, IH component 2 variance and heart failure likely represent the net effect of multiple complex, interactive relationships connecting the heart to red cells and lymphocytes (Fig. 4). Additional research is needed to identify the operative pathways most relevant to outcomes.

Like heart failure, diabetes is considered a pro-inflammatory condition with altered cellular metabolism and oxidative stress. Diabetes is known to result in defective neutrophil activation and impaired production of reactive oxygen species^27,28^. Like heart failure, diabetes is also associated with systemic TNFα elevation, derived in part from paracrine effects of adipose tissue^29^.

Few studies have analyzed the relationships between diabetes and immunohematologic homeostasis at the population level, although a recent report using machine learning identified age and total leukocytes as the most important predictors of diabetes^30,31^. Consistent with this, we find diabetes to be associated with greater leukocytosis compared with non-diabetics (i.e. IH component 1), and this was independent of other potential confounders. This leukocytosis was similar in magnitude to that observed in relation to heart failure. Moreover, the extent of anemia was similar in diabetes and heart failure. Compared to heart failure, diabetes was associated with a lower RDW, and instead of lymphopenia as in heart failure, diabetes was associated with lymphocytosis.

Diabetes was associated with substantial COVID-19 mortality risk, but (unlike heart failure) this risk was only minimally explained by its associated IH variation. Our interpretation of these data is that the COVID-19 risk associated with diabetes derive from a set of mechanisms distinct from heart failure and poorly reflected by IH parameters. Deeper biomarker discovery efforts and analyses of the effects of various medications are needed to identify more precise correlates and reversible mechanisms of diabetes-associated COVID-19 risk.

This study included a large sample size, allowing for greater precision in the estimates of the association between CVD and COVID-19, and allowed for adjustment for many potential confounders, but residual confounding is possible. We restricted our analysis to those with an available CBC, potentially biasing results to reflect those with greater pre-pandemic contact with healthcare. The characterization of comorbidities was based upon the use of ICD-9/10 diagnoses which can be prone to misclassification. The presence of an EF value was based upon reporting within the VA and potentially missed patients receiving care in another healthcare setting. As an electronic health record-based study, our results may be affected by other sources of missingness, misclassification, unmeasured effects, and/or residual confounding. Statin usage was based upon prescription data and medical compliance could not be verified. Other medications were not accounted for and the potential immunologic effects of common cardiovascular medications represents an important area of future study. We assumed that death within 60 days of COVID-19 was infection-related which may overestimate certain associations. The extent to which these findings hold in the post-vaccination era was not addressed in this report. Whether mediating IH factors reflect pathways which are cause, effect, or epiphenomenal to heart failure or COVID-19 risk will require additional studies. The extent to which the cytokine levels reflect the influence of COVID-19 infection is unknown. The IH parameters used in this study are crude, but these findings provide critical population level insight into heterogeneity in immune function.

In conclusion, we find that heart failure and its associated IH alterations are anticipatory indicators of COVID-19 mortality risk. These findings highlight the need for a deeper understanding of the processes (e.g. immunologic, metabolic, neurohormonal, hematopoietic, and circulatory) and heterogeneity thereof which link heart failure, dysregulated immune and hematologic homeostasis, and resilience to infection.

## Methods

### Study design

This was a retrospective-prospective cohort study of the Veterans Health Administration (VHA) population to analyze relationships between IH parameters (i.e. CBC/differential values and their principal components), prevalent co-morbidities, CVD risk factors, and COVID-19 mortality. A biomarker analysis was performed in a cohort of patients receiving care at the Louis Stokes Cleveland Veterans Affairs Medical Center (LSCVAMC). The study was approved by the Institutional Review Board of Case Western Reserve School of Medicine and the LSCVAMC.

### Study populations

The study population included United States Veterans receiving care through the VHA who (1) were diagnosed or treated for SARS-COV2 in the VHA between March 1, 2020 through December 31, 2020, and (2) had an outpatient CBC less than 1 year and more than 1 week prior to the index positive SARS-COV2 polymerase chain reaction (PCR) test date (*n* = 92,533), the date of which serving as study baseline. The first wave of COVID-19 was selected to avoid the confounding influence of vaccination usage and differences in the pathogenicity of subsequent viral strains. Data were extracted from the VA Corporate Data Warehouse and the VA COVID-19 Shared Data Resource through the VA Informatics and Computing Infrastructure. Separately, LSCVAMC outpatients from the Cleveland VA who were more than 3 months removed from a positive SARS-CoV2 test during this first wave of COVID-19 were recruited and consented for blood procurement (*n* = 44).

### Study variables

Demographic and clinical predictors included age, sex, race, ethnicity, systolic blood pressure (SBP), diastolic blood pressure (DBP), total cholesterol (TC), high density lipoprotein (HDL) level, low density lipoprotein (LDL) level, body mass index (BMI), serum creatinine (Cr), blood urea nitrogen (BUN), statin prescription, and the following prevalent (pre-COVID-19) clinical co-morbidities at study baseline: prior myocardial infarction (MI), heart failure, diabetes, pulmonary disease, tobacco use disorder, rheumatologic disease, and cancer were identified using ICD-9/10 codes. Diabetes in this VA population is overwhelmingly adult onset since juvenile diabetes has historically precluded enlistment in the military. In those with a diagnosis of heart failure, the most recent left ventricular ejection fraction (EF) by echocardiogram prior to COVID-19 was obtained using a VA database of values extracted from echocardiogram reports using the natural language processing model originally derived by Patterson et al^32^. This tool extracts EF values from text integration utilities documents including history and physical examination notes, progress notes, discharge summary notes, echocardiography reports, nuclear medicine reports, cardiac catheterization reports, and other cardiology notes. COVID-19 death was defined as death within 60 days of positive SARS-COV2 testing. Death and time to event was ascertained using combined VHA, Social Security, and Center for Medicare and Medicaid Services data. The last patient encounter within the VHA was retained and, if the last encounter was less than 60 days after the index COVID date, the follow up time was censored at that encounter date.

Individual immunohematologic (IH) parameters were the following 10 components of the CBC panel with differential leukocyte counts: absolute lymphocyte count (ALC), absolute monocyte count (AMC), absolute neutrophil count (ANC), absolute eosinophil count (AEC), absolute basophil count (ABC), red cell distribution width (RDW), hemoglobin (HGB), platelet count (PLT), mean platelet volume (MPV) and mean corpuscular volume (MCV). Where an individual lab parameter was unreported, imputed values were used based on the present CBC parameters using imputePCA within the missMDA package in R. Since immunologic mechanisms are expected to impact multiple individual parameters, a principal components analysis (PCA) of these 10 CBC parameters was performed using Varimax rotation with Kaiser Normalization to extract components with eigenvalues greater than 1. These components represent the dimensions across which the greatest degree of shared variation involving the 10 CBC parameters, collectively, are expressed. Factor scores were used as continuous predictor variables in unadjusted and adjusted regression models.

### Statistical methods

Standard univariable summary statistics, including means and standard deviations for continuous variables and proportions for categorical variables, were used to describe baseline characteristics. IH factors were expressed as standardized values to facilitate cross-comparisons among these parameters. Parameters with protective associations with mortality were inverted in these analyses to facilitate cross-comparisons with co-morbidities. IH factors were additionally analyzed as categorical features, using quintiles as cut points to define strata.

The relationship between prevalent co-morbidities (or IH parameters) and COVID-19 mortality was assessed using Cox proportional hazards regression to model time from positive SARS-CoV2 testing to death within 60 days. We estimated 1) unadjusted models, 2) models adjusted for age, sex and race/ethnicity (demographically adjusted), 3) and adjusted models which additionally included diabetes mellitus, heart failure, SBP, DBP, TC, HDL, LDL, Cr, BUN, BMI, tobacco use, prior MI, pulmonary disease, rheumatologic disease, cancer, statin prescription with or without IH components. We used multivariable logistic regression to identify the IH characteristics which accompanied prevalent co-morbidities prior to COVID-19. In addition to heart failure and diabetes, tobacco use, prior MI, pulmonary disease, rheumatologic disease, and cancer were similarly analyzed for context. Mediation analyses were performed to estimate the direct and indirect (via IH components) relationships between prevalent heart failure (or diabetes) and COVID-19 mortality. The PROCESS (v4.0) plugin for SPSS was used to report beta coefficients corresponding to indirect effects (via the 3 IH components) versus direct effects linking comorbidity to COVID-19 mortality, fully adjusting for the socio-demographics and clinical features.

### Post-COVID-19 antibody and cytokine analysis

SARS-CoV2 specific antibody levels were quantified as previously described^33^. Cytokine levels were measured using freshly thawed aliquots of cryopreserved plasma in batch with the MesoScale Discovery multiplex (U-plex) platform. Comparisons were performed using spearman correlations. Antecedent IH components and post-infection IL-6 was also analyzed using linear regression models adjusting for age, sex, race/ethnicity, and time from positive SARS-CoV2 testing.

### Software

Statistical analyses were performed using R Version 4 (R Foundation for Statistical Computing, Vienna Austria) and SPSS version 28. Additional visual outputs used graphpad prism 10 software and BioRender applications.

## Supplementary information

Supplementary Material

## Data Availability

Requests to be considered in accordance with policies of the United States Veterans Health Administration.
