# Supplementary material for: Pre-exposure immunohematologic features of heart failure associate with COVID-19 mortality
Source: NPJ Cardiovasc Health. 2024 Nov 21;1:32. doi: 10.1038/s44325-024-00025-7 (PMC12912350; doi:10.1038/s44325-024-00025-7)
Supplement: Supplementary file 1 — Supplementary Material [file 44325_2024_25_MOESM1_ESM.docx]

Pre-exposure Immunohematologic Features of Heart Failure Associate with COVID-19 Mortality

Brief Title: Pragmatic Indicators of Heart Failure-related Immunocompromise

*David A. Zidar, MD PhD^1,2,3^; Brigid M. Wilson, PhD^2^ ; Sadeer G. Al-Kindi, MD^1,3^; David Sweet MD PhD^1^; Steven Juchnowski^1^; Lauren Huntington^2^; Carey Shive PhD^1,2^; Jurgen Bosch, PhD MBA^1^; Christopher King MD^1,2^; Jonathan Karn, PhD^1^; Mina K. Chung MD^4^; Carl B. Gillombardo^1,3^, MD; Mohammad Karnib, MD^1,2^; Varun Sundaram MD^1,2,3^; Sahil A. Parikh MD^6^; Mukesh Jain, MD^7^; Douglas D. Gunzler, PhD^5^; Jacek Skarbinski MD^8^; W. H. Wilson Tang MD^4^; Donald D. Anthony MD PhD^1,2^; Timothy Chan, MD PhD^4^; Jarrod E. Dalton, PhD^4^*

**Supplemental Results**

Supplemental Table 1 ……………………………………………………………...………… page 2

Supplemental Table 2 …………………………………………………………………….….. page 3

Supplemental Table 3 …………………………………………………………………...…… page 4

Supplemental Figure 1 ...………………………………………………...…...………………. page 5

Supplemental Table 4 ..………………………………………………………………….…… page 6

Supplemental Figure 2 .…………………………………………………………………….…. page 7

Supplemental Table 5 .………………………………………………………………….……. page 8

Supplemental Figure 3 .…………………………………………………………………….…. page 9

Supplemental Table 6 ……………………………………….………………………….....…. page 10

**Supplemental Table 1. Co-morbidities and Risk of Death after COVID-19**

**Supplemental Table 2. Rotated Component Matrix from Principal Components Analysis of Complete Blood Cell Count Variables.**

**Supplemental Table 3. Association of Pre-COVID-19 Immunohematologic Features and COVID-19 Mortality**

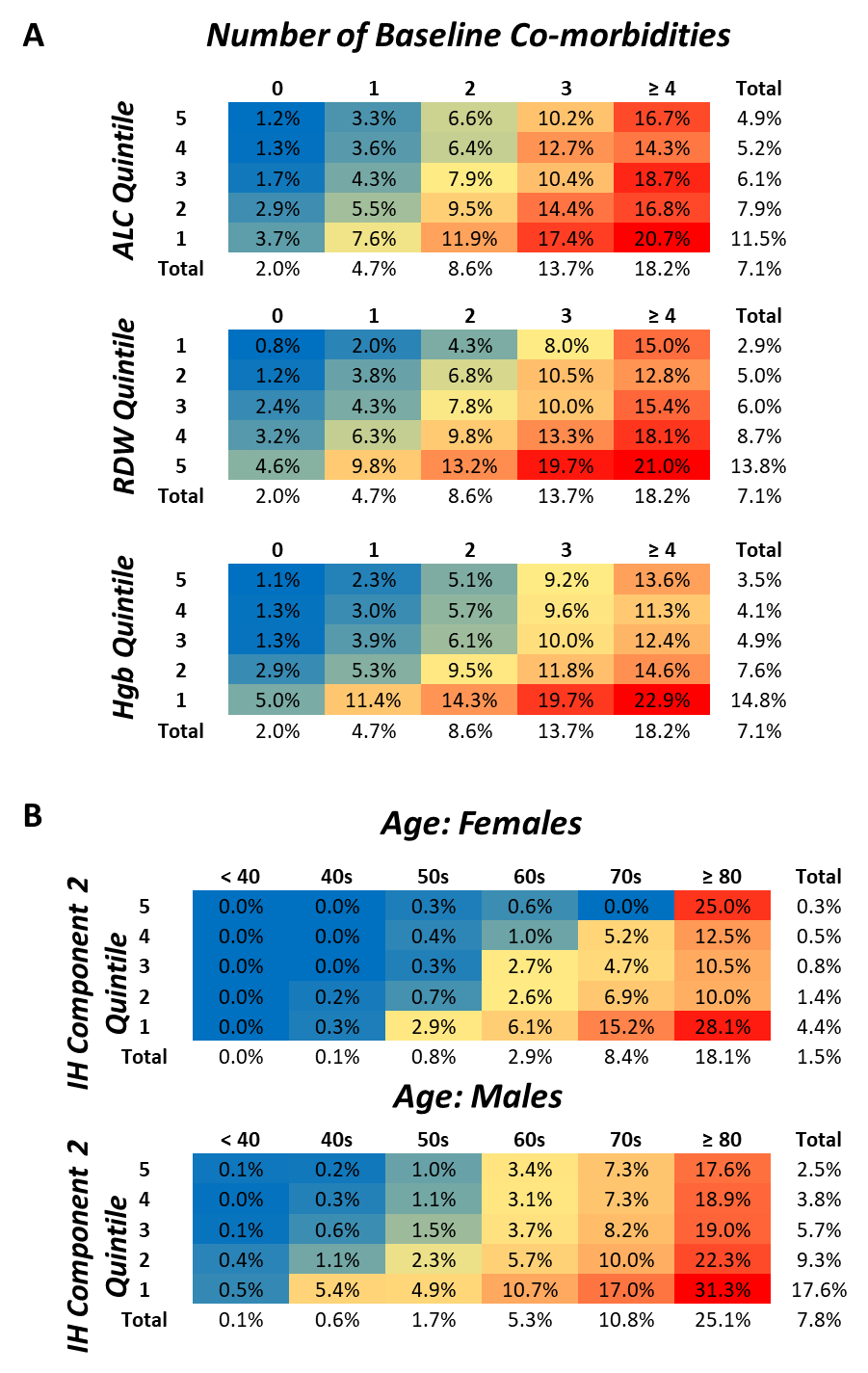


**Supplemental Figure 1. Immunohematologic Parameter Variance and COVID-19 Mortality Across Co-Morbidity and Age Strata. (A)** Crude 60 day mortality rates (as percentages and heat map: red higher than blue) after positive SARS-COV2 testing are shown in relation to the quintile of absolute lymphocyte counts (ALC), red cell distribution width (RDW), or hemoglobin (Hgb) as shown (rows) and the number of comorbidities (diabetes, heart failure, prior myocardial infarction, pulmonary disease, rheumatologic disease, tobacco use disorder, cancer). **(B)** 60 day mortality rates (as percentages and heat map: red higher than blue) after COVID-19 are shown in relation to the quintile of Immunohematologic (IH) component 2, and the decade of age in males and females (columns).

**Supplemental Table 4. Demographics and Characteristics of Immune Analysis Cohort**

**
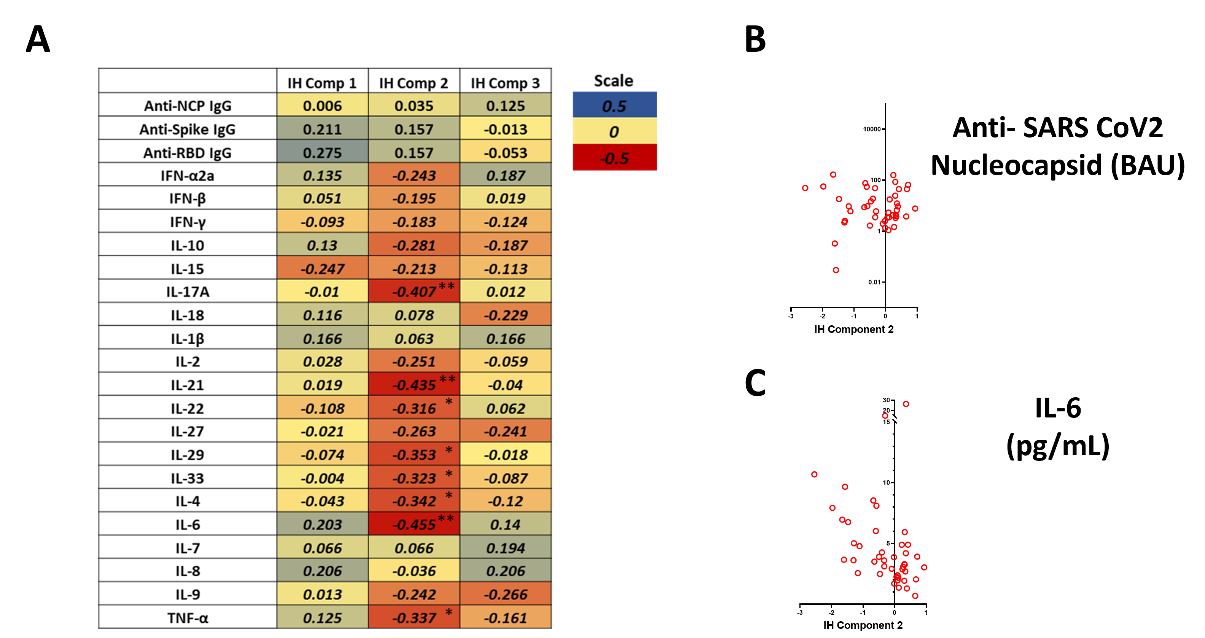
**

**Supplemental Figure 2. Post-Recovery Inflammation is Related to Pre-COVID-19 Immunohematologic (IH) Component 2.**  SARS- CoV2 specific antibody titers and plasma cytokine levels from patients (N=44) convalescent from COVID-19 were analyzed in relation to pre-COVID-19 immunohematologic (IH) components. Spearman rho values are shown and this strength of association depicted as a heat map (blue: positive association; red: inverse association). (*: p<0.05; **: p<0.01).

(NCP: nucleocapsid; RBD: receptor binding domain; IFN: interferon; IL: interleukin; TNF: tumor necrosis factor)

**Supplemental Table 5. Association Between Variance in Immunohematologic Parameters and Components and a Heart Failure Diagnosis Prior to COVID-19**

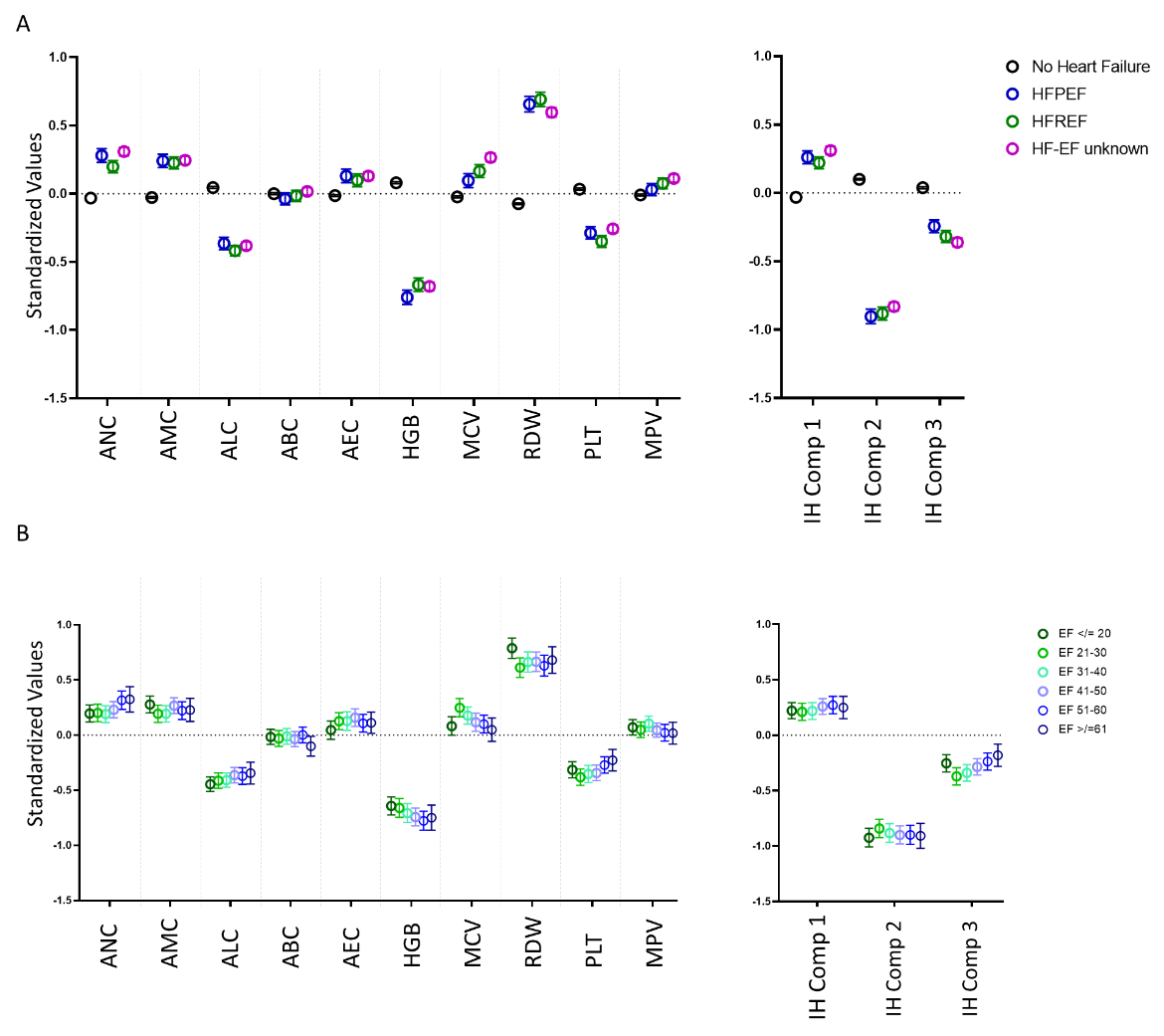


**Supplemental Figure 3. Immunohematologic Characteristics of Heart Failure According to Ejection Fraction. (A)** Standardized IH variables (mean, 95% CI) are shown for those without heart failure (black, n= 82,927), those with preserved ejection fraction heart failure (blue, n=2001), those with reduced ejection fraction heart failure (red, n = 2,434), and heart failure/missing EF (pink, n=5,171). **(B)** Among those with heart failure and known EF, standardized IH features are shown according to EF group: >60 (blue), 50-60 (dark green), 40-50 (red), 30-40 (orange), 20-30 (brown), <20 (green). (Hemoglobin: HGB; Mean corpuscular volume: MCV; Red cell distribution width: RDW; Absolute neutrophil counts: ANC; Absolute monocyte counts: AMC; Absolute lymphocyte counts: ALC; Absolute basophil counts: ABC; Absolute eosinophil counts: AEC; platelet counts: PLT; mean platelet volume: MPV)

**Supplemental Table 6. Mediation Analysis Comparing Immunohematologic Components As Correlates of Co-morbidity Specific COVID-19 Morality Risk**
